# Knowledge, Perceptions and Concerns of Diabetes-Associated Complications among Individuals Living with Type 1 and Type 2 Diabetes Mellitus

**DOI:** 10.3390/healthcare8010025

**Published:** 2020-01-30

**Authors:** Clara Sanz-Nogués, Mohamad Mustafa, Helen Burke, Timothy O’Brien, Cynthia M. Coleman

**Affiliations:** 1 Regenerative Medicine Institute, Biomedical Science Building, National University of Ireland, Galway H91 W2TY, Irelandtimothy.obrien@nuigalway.ie (T.O.); 2 Diabetes Center, University Hospital Galway, Saolta University Health Care Group, Galway H91 YR71, Ireland; dr.mohamadjaffer@yahoo.com (M.M.); helen.burke@hse.ie (H.B.)

**Keywords:** type 1 diabetes, type 2 diabetes, diabetes complications

## Abstract

The purpose of this study was to investigate the knowledge, perceptions and concerns of individuals living with diabetes mellitus regarding the disorder and its associated long-term health complications. Individuals living with type 1 (*N* = 110) and type 2 (*N* = 100) diabetes were surveyed at the Diabetes Centre at University Hospital Galway (Ireland). A questionnaire was used to record respondent’s perceptions and concerns about living with diabetes and developing associated long-term health complications, especially diabetes-induced osteopathy. Participants’ responses revealed a variety of perspectives. Individuals with type 1 diabetes had a deeper understanding of the aetiology of diabetes and were more concerned about its complications than individuals with type 2 diabetes. The most recognized complications identified by the participants were retinopathy (92% type 1; 83% type 2), amputations (80% type 1; 70% type 2) and nephropathy (83% type 1; 63% type 2). Diabetes-related osteopathy was under-recognized, with 37% (type 1) and 23% (type 2) of respondents identifying bone fractures as a diabetes-related complication. Enhancing the patient awareness of this under-recognized diabetes-associated complication and ensuring that preventative measures are incorporated within health care programmes may offer methodologies to address this complication clinically.

## 1. Introduction

Diabetes mellitus represents a heterogeneous group of chronic metabolic disorders characterized by hyperglycaemia resulting from impaired insulin secretion or action [1]. Poorly controlled diabetes is associated with short- and long-term health complications, which commonly result in hospitalization, disability, a lowered quality of life and early death. In addition, it imposes a significant burden to healthcare systems, on individuals living with diabetes and their families [1]. 

Bone disease, or osteopathy, has been highlighted as a long-term complication of diabetes [2,3], and can be prevented and/or managed during all stages of life [4]. In particular, long-term exposure to the diabetic milieu elicits changes in bone metabolism and bone microarchitecture, which result in an increased risk of fracture and decreased healing rates [2,5,6]. Hip fractures are a significant basis of disability and mortality in middle-aged and older adults [7]. Previous studies have shown that both men and women with type 1 diabetes have low bone mineral density and an increased fracture risk [8,9,10]. Type 2 diabetes has been associated with normal or increased bone mineral density, and paradoxically, increased fracture risks, after adjusting for multiple covariates including frequency of falls [10,11,12,13]. The association between the type of diabetes and hip fracture incidence is stronger for type 1 diabetes (summary relative risk [RR] 6.3) than for type 2 diabetes (summary RR 1.7) [14]. Similarly, in another meta-analysis conducted by Vestergaard, the risk ratio for diabetes and hip fracture was 6.9 for type 1 diabetes and 1.38 for type 2 diabetes [15]. Nevertheless, the mechanisms whereby diabetes increases fracture risk are not entirely clear, and it seems to differ among the two types of diabetes [2,5,15]. 

While the link between diabetes and bone disease is well-recognized in the scientific community, with abundant published literature reviewed above, there is still limited knowledge about diabetes-related osteopathy in individuals living with diabetes [16,17]. Indeed, there is little evidence available about its optimal management [5], and clinical guidelines do not include specific recommendations [18,19].

To date, there are limited studies that have investigated the current knowledge, perceptions, and concerns of people living with diabetes of this disease and its long-term health complications. Some studies reported lack of public awareness and knowledge on various aspects related to diabetes [20,21]. Several reports have shown that a significant percentage of people living with diabetes were unaware of potential diabetes-related complications [22,23]. An Irish study has shown that knowledge is a key parameter for individual’s self-management of diabetes [24]. In addition, having a comprehensive understanding of patients’ concerns, and the social, physiological and health-related impacts of diabetes on the population is important for informing policy and practice in the healthcare system. The aim of this study is to understand the current knowledge, perceptions, and concerns of individuals living with diabetes about the disease and its long-term health complications, with a particular focus on awareness of diabetic-related osteopathy.

## 2. Materials and Methods 

### 2.1. Participant Recruitment and Sampling

The survey consultation took place between February to April 2017 and March to May 2018 at the Diabetes Centre at University Hospital Galway (UHG). Ethical approval for this study was granted by the Galway University Hospital Research Ethics Committee (C.A. 1535). Participants’ eligibility criteria were predefined to include adults of at least 18 years of age who were residents in Ireland at the time of the study and who had a clinical diagnosis of diabetes for greater than 6 months. Participants were approached randomly as they were entering the waiting room at the Diabetes Centre at UHG, and received a patient information brochure, consent form and a questionnaire to be self-administered. Participants were included in the study only after informed consent and were guaranteed confidentiality, adhering to the principles of the Helsinki Declaration [25]. 

Overall, 234 questionnaires were distributed. A total of 222 participants returned the questionnaires (participation rate 94.9%). From these, 7 questionnaires were excluded based on an unsure or early (< 6 months) diagnosis of diabetes; 5 questionnaires were considered invalid (respondents did not complete at least the basic profiling questions and/or left the majority of questions blank). The final sample was therefore comprised of 210 participants: 110 with type 1 diabetes and 100 with type 2 diabetes (Table 1). Although researchers approached participants at random, the presence of selection bias cannot be completely discarded. As the data collected from non-participants were not included in the analysis, it is possible that inherent self-selection differences between non-participants and participants could contribute to a survey bias.

### 2.2. Questionnaire 

The questionnaire was designed in English and addressed the following topics: A) Demographics (Irish county of residence, age, gender, level of education) and clinical data (diabetes type, source of diagnosis, years following diagnosis); B) What is diabetes? Describe it or give phrases associated with diabetes (self-generated responses); C) A 10-point analogue scale, where [0] = no concerns and [10] = extreme anxiety about developing potential complications of diabetes to ascertain the participant’s level of concern about developing potential complications of diabetes; D) List concerns regarding potential complications of diabetes (self-generated response); E) Knowledge of associated complications assessed by the respondents’ ability of identifying diabetes-related complications from a list of 15 words. They were asked to circle all that applied to their knowledge [15 Words: 1. Blindness; 2. Stroke; 3. Foot ulcers; 4. Foot/Hand numbness; 5. Dialysis; 6. Asthma; 7. Heart disease; 8. Amputations; 9. Digestion difficulties; 10. Kidney failure; 11. Increased infections; 12. Bone fractures; 13. Liver failure; 14. Difficulty conceiving; 15. Skin rashes]; F) Describe how diabetes can affect bone health or the ability to heal bone fractures (self-generated response); G) Identify their primary source of information about diabetes (choose from Family doctor, Diabetes specialist, Endocrinologist, Internet, Eye doctor, Podiatrist, Books/magazines, Diabetes support groups or Community nurse). The majority of questions requested open-ended responses, allowing for self-expression and to obtain more in-depth understanding of participant’s knowledge or perceptions about the topic. The questionnaire was designed by the authors of this study, a collaboration between scientists and healthcare professionals working at the Diabetes Centre and does not correspond to any questionnaire on diabetes-related knowledge validated in the Irish population. Prior to starting the study, the questionnaire was piloted with a small sample of volunteers living with diabetes to refine the contents and clarity of the questionnaire. The final questionnaire was self-administered, but participants were able to ask questions or receive help from the researcher if needed. Some participants (<1% = type 1; 28% = type 2) reported personal limitations such as impaired vision, hand tremors or literacy difficulties. In these cases, the investigator served as a scribe. 

### 2.3. Statistical Analysis

Qualitative data and self-generated answers were analyzed and grouped into common themes or a core category. Responses were transformed into quantitative data when possible for statistical analysis, by counting the frequency of appearance of the core theme or category among the responses. Differences among individuals living with type 1 or type 2 diabetes were evaluated using a 2-sample t-test for continuous variables. Association among categorical variables was studied using Pearson’s Chi-square analysis. The difference in proportion among respondents living with type 1 or type 2 diabetes was assessed using a 2-sample interval estimate for the difference in population proportions for each category. Multivariate regression analysis was used to investigate potentially confounding predictors of participant’s concerns and disease knowledge, such as age, years following diagnosis, gender and level of education. All statistical tests were two-tailed and statistical significance was considered as *p*-value of ≤0.05. Statistical analysis of data was performed using the Minitab 17 Statistical Software (Minitab, State College, PA, USA).

## 3. Results

### 3.1. Participants’ Characteristics

The socio-demographic and clinical characteristics of participants are shown in Table 1. The mean age of individuals living with type 1 diabetes (43 ± 14 years) attending the Diabetes Centre at UHG was significantly lower than that of those individuals with type 2 diabetes (69 ± 11 years) (*p*-value < 0.001). The differences observed in the participants’ reported personal limitations when self-administering the questionnaire (<1% = type 1; 28% = type 2) are most likely attributed to this difference in age between groups (i.e. more personal limitations with age). 

Respondents with type 1 diabetes received their diagnosis an average of 21 ± 13 years prior, while individuals with type 2 diabetes had lived with the condition for an average of 11 ± 6 years before participating in this study. The majority of participants lived in Galway (type 1 = 79%; type 2 = 83%). A significantly different percentage of individuals with type 2 diabetes (25.5%) completed school following a primary level of education compared to 2.7% in the type 1 diabetes group (*p*-value < 0.001). In contrast, more individuals with type 1 diabetes had pursued further education, including 30% completing an undergraduate degree and 19% a post-graduate degree compared to 13% (*p*-value 0.002) and 9% (*p*-value 0.037) in the type 2 diabetes group, respectively. The education level differences observed are likely due to the age differences among both groups (Supplementary S1).

### 3.2. Participants’ Viewpoint Regarding Living with Diabetes

Participants were asked to define diabetes and/or to give phrases associated with diabetes in an open-ended question. The responses were grouped into common trends where the participants’ answers were quantified according to the presence of specific key words or themes. When a response contained more than one word or theme, the count was included in the 2 (or more) different categories. The non-response rate for this question was 10% in the type 1 diabetes group and 9% in the type 2 diabetes group (Supplementary S2). 

Overall, participants with type 1 diabetes provided more sophisticated responses (>2–3 lines), often including specific scientific terminology such as ‘*beta cells*’, ‘*Islets of Langerhans’*, ‘*autoimmune’* or ‘*immune reaction’*, which may reflect a higher level of biologic understanding on the aetiology of the disorder. There was a tendency for participants to give a definition of diabetes according to the type of diabetes with which they live. For example, the majority of respondents living with type 1 diabetes defined diabetes as a ‘*disease in which the pancreas stops making insulin’* (74.5%), while participants with type 2 diabetes most frequently defined diabetes as ‘*a disease in which there are elevated levels of sugar in the blood*’ (39%). In 4% of the responses given by participants with type 1 diabetes, a description of type 2 diabetes was also included while the reverse was not observed. 

Other noteworthy differences were observed among the answers given by respondents with type 1 and type 2 diabetes. Individuals living with type 2 diabetes highlighted complications of diabetes (e.g. kidney damage, loss of eyesight, foot ulcers and amputations) in 7% of their responses, as compared to 2% in the cohort living with type 1 diabetes. There was a considerable percentage of responses in both type 2 diabetes group (17%) and type 1 diabetes (9%), that related ‘diabetes’ with words associated with challenges and/or negative thoughts, such as ‘*annoying’*, ‘*a pain in my…*’, ‘*frustrating’* or ‘*painful’*. These responses may underline a level of anxiety and/or suffering in individuals living with diabetes.

### 3.3. Participants’ Level of Concern Regarding Developing Long-Term Diabetes-Related Complications

Participants were asked to indicate their level of concern regarding developing potential complications of diabetes using a 10-point visual analogue scale (Table 2). Overall, individuals living with type 1 diabetes were more concerned than those with type 2 diabetes, with a median (IQR) level of concern of 6.5 (4 to 8) and a mode of 7 in the group with type 1 diabetes and a median (IQR) of 5.5 (5 to 7) and a mode of 5 in the group with type 2 diabetes. There was a significantly higher percentage of participants with type 2 diabetes (15 out of 98) who responded having a level of concern between 0 to 2, compared to 7 out of 110 participants in the type 1 diabetes group (*p*-value = 0.038). 

Multivariate linear regression analysis was performed to assess the association between four confounders (age, years following diagnosis, gender and level of education) and respondents’ level of concern about developing complications of diabetes (Supplementary S3). This analysis showed that age, gender, and level of education was not significantly associated with the level of concern in respondents with type 1 and type 2 diabetes (*p*-value > 0.05). Nevertheless, the time following a diagnosis of diabetes had a significant relationship with the level of concern in participants with type 2 diabetes (regression coefficient 0.125; 95% CI 0.035, 0.215) (*p*-value 0.007) but not in participants with type 1 diabetes (regression coefficient 0.031 95% CI −0.009, 0.070) (*p*-value 0.126).

Participants were then asked to list, in an open-ended response, what concerned them the most. Participants’ responses were analysed and quantified according to common themes or categories (Table 2). In general, individuals with type 1 diabetes were more concerned about blindness (69%), circulation problems such as foot ulcers and amputations (41%) and kidney disease (63%). The majority of individuals with type 2 diabetes were more concerned about foot ulcers and amputations (49%), followed by blindness (43%), but were less concerned about developing kidney disease (10%). In addition, 14% of participants with type 2 diabetes specified having no concerns about developing diabetes-related complications, which was significantly higher than the percentage in the group with type 1 diabetes (4%). Interestingly, only 3 out of 110 participants living with type 1 diabetes and 2 out of 100 living with type 2 diabetes highlighted concerns about developing bone problems and/or osteoporosis. This denotes a low level of concern regarding developing osteopathy as a result of diabetes, which may be due to a low level of awareness regarding this diabetes-associated complication. 

### 3.4. Participants’ Knowledge Regarding Diabetes-Related Complications

Participants were provided with a list of 15 words representing potential health complications of diabetes and were asked to circle all of the words that applied. The total number of complications identified per participant was recorded (Table 3). Results showed that individuals with type 1 diabetes identified more diabetes-related complications, with a median (IQR) of 9 (6 to 11) complications, compared to a median (IQR) of 6 (4 to 9) complications identified in the type 2 diabetes group (*p*-value 0.001). Multivariate linear regression analysis was performed to assess the association between four confounding variables (age, years following diagnosis, gender and level of education) and the disease knowledge, estimated by the number of complications of diabetes identified by participants with type 1 and type 2 diabetes (Supplementary S3). The results indicated that none of the four variables were independently associated with the number of complications identified by the respondents (*p*-value > 0.05).

The identification frequency of each diabetes-associated complication was calculated (Table 3). Note that the presence of the word ‘dialysis’ and/or ‘kidney damage’ counted as only one category called ‘nephropathy’. Similarly, the presence of the words ‘amputations’ and/or ‘foot ulcers’ were also counted as one category. Results showed that retinopathy (blindness) was the most frequently identified diabetes-related complication by both participants with type 1 diabetes (92%) and type 2 diabetes (83%). In the group of individuals with type 1 diabetes, this was followed by nephropathy (dialysis and/or kidney damage) (83%), amputations and/or foot ulcers (80.5%) and heart disease (72%). In the cohort with type 2 diabetes, amputations and/or foot ulcers (70%), nephropathy (63%) and heart disease (62%) followed frequency. All of these are very well-known long-term health conditions related to diabetes. Other less well-known diabetes-related health conditions such as pregnancy difficulties, skin rashes, and digestion difficulties were less frequently identified by both individuals with type 1 and type 2 diabetes. 

Diabetes-induced osteopathy, represented as ‘bone fractures’ in the list, was identified in 37% (type 1 diabetes) and 23% (type 2 diabetes) of the surveys. As women have a higher risk of bone fracture [26] and have previously been shown to have an increased awareness of diabetes-induced osteopathy [16], a univariate binary logistic regression analysis was used to assess the association between gender and the appearance of ‘bone fracture’ in the responses of participants with type 1 and type 2 diabetes. The results showed that gender was not significantly associated with selecting ‘bone fracture’ as a complication of diabetes in participants with type 1 (odds ratio 0.7935; 95%CI 0.362, 2.744) (*p*-value 0.565) and type 2 diabetes (odds ratio 0.477; 95%CI 0.1852, 1.227) (*p*-value 0.125).

### 3.5. Participants’ Understanding of Diabetes-related Osteopathy

To examine the participants’ knowledge or awareness of diabetes-osteopathy, participants were asked to describe how they thought diabetes could impact their bone health. Individuals with type 1 diabetes provided more elaborate responses (>2–3 lines), with a greater variety of proposed cellular or molecular mechanisms of how diabetes could affect their bone health (Supplementary S4). A significant number of participants in both cohorts either did not respond (type 1 = 17%, type 2 = 21%), were unsure or unaware of this topic (type 1 = 34.5%, type 2 = 40%), or gave inaccurate or unrelated answers to the question (type 1 = 6%, type 2 = 8%), highlighting a poor level of awareness linking bone health to diabetes. The majority of individuals who responded suggested that diabetes influenced bone health by increasing healing times and/or decreasing bone strength (type 1 = 27%; type 2 = 26%); weakening the immune system and/or increasing the risk of infections (type 1 = 8%; type 2 = 5%), or decreasing circulation (type 1 = 4.5%; type 2 = 2%). Individuals with type 1 diabetes mentioned other mechanisms such as compromised bone cells (2%) or vitamin D deficiency (4.5%).

## 4. Discussion

The results from this study revealed a complex picture of the awareness of diabetes and its long-term complications among respondents living with type 1 and type 2 diabetes. Respondents with type 1 diabetes generally provided sophisticated responses and included scientific terminology when defining the term diabetes and/or describing living with diabetes as compared to those living with type 2 diabetes. This may reflect a deeper understanding of the disease in this cohort. A significant number of respondents in both cohorts emphasized living with the disorder as being ‘*life-altering’,* ‘*frustrating’*, ‘*a pain’*, ‘*annoying’* and ‘*hard work’*. These responses may highlight a level of distress or regarding living with this condition. Emotional distress specifically related to living with the demands of diabetes, termed ‘diabetes distress’, has been reported in other studies [27]. Diabetes distress encompasses distress related to diabetic regimen (struggles with self-management of the disease), interpersonal issues (feeling unsupported in self-management efforts), relationship with healthcare professionals (feelings about care and information provided) and emotional burden (feelings such as hopeless or failure when thinking about the disease) [27]. The presence of diabetes distress is an important finding in this study, and it should not be overlooked. Disease-related distress have previously shown to be a stronger predictor of poor glycaemic control [27]. This is concordant with other studies, which showed that interventions that aimed at reducing regimen-related distress are predictive of improved future glycaemic control [28,29]. Moving forward, an evaluation of diabetes distress should be incorporated into all clinical visits and healthcare plans.

Understanding the concerns and priorities of individuals living with diabetes is critical to public and patient involvement (PPI) in research. PPI ensures that A) patients are informed of research that is relevant to them, generating increased patient support for engagement in research, B) ensures patient experiences are considered when making research decisions and C) encourages engagement to sculpt research questions to areas most relevant to the patient, improving the relevance of the research outcomes [30]. By asking patients what aspects of living with diabetes are of the most concern, clinicians and research scientists can identify and prioritize research areas ensuring the greatest impact on those living with the disease. In this study, it was identified that individuals with type 1 diabetes were more aware of, and concerned about, developing potential long-term complications of diabetes than those with type 2 diabetes. The differences in the responses found between these two cohorts may be explained by several factors. Individuals with type 1 diabetes are a younger population (43 ± 14 years.) with more educational opportunities as compared to individuals with type 2 diabetes (69 ± 11 years.) (Supplementary S1). However, results from the multivariable linear regression analysis showed that the level of education was not significantly associated with the level of concern reported by individuals with type 1 and type 2 diabetes (Supplementary S3). Alternatively, individuals with type 2 diabetes often reported co-morbidities associated with aging, such as cancer or cardiac disease, and therefore demonstrated a reduced focus on diabetes and its associated complications. Interestingly, the level of concern increased significantly with the time following diagnosis in individuals with type 2 diabetes, but not in the cohort with type1 diabetes (Supplementary S3).

The level of disease knowledge was assessed by the participant’s ability to recognize diabetes-related complications from a list. Results showed that individuals with type 1 diabetes were able to identify more diabetes-associated complications compared those living with type 2 diabetes, and that the level of knowledge was not significantly associated with participant’s age, gender, level of education or years following diagnosis. Overall, well-known diabetes complications such as retinopathy, nephropathy, circulation problems, and heart disease were the most frequently identified. 

A focus of this study was to explore the level of recognition and understanding of diabetes-related osteopathy, an often under-recognized complication of diabetes. This study revealed a low level of awareness and understanding of bone-associated complications of diabetes when participants were asked to A) identify bone fractures or delayed bone healing from a list of potential complications or B) discuss how diabetes may impact bone health. Other published studies have also explored the knowledge of diabetes-related complications in people living with diabetes [20,21,22,23], some focusing on assessing a participant’s understanding of diabetic osteopathy [16,17]. The trends identified in this study are comparable to those identified internationally, which is interesting given the multivariate nature of the understanding potentially cofounded by age, gender, geographical localization, availability of educational programmes, country-dependent management of diabetes care, and other societal factors.

### 4.1. Clinical Implications 

With the notably increased prevalence of diabetes, practical approaches are necessary to prevent or significantly delay devastating diabetes-related complications as well as to increase an individual’s quality of life. With appropriate knowledge and disease management, individuals living with diabetes can directly influence their health with lifestyle changes including physical activity and nutrition. This study revealed a different level of concerns and knowledge/awareness of diabetes and its long-term complications among individuals living with type 1 and type 2 diabetes, suggesting that different approaches may be needed to address this condition clinically.

In addition to this, we identified a low level of awareness and understanding of diabetes-induced bone disease. Osteopathy is often preventable and/or manageable, and with proper guidance and support from the healthcare providers, individuals with diabetes can directly influence their bone health at any stage of their life. Well-designed population-based interventions, community projects, and media interventions can be highly effective in preventing disease, injury, disability and premature death [4]. Results from Irish educational programmes have shown a gain of diabetes-related knowledge and importantly, an association with a sustained clinically significant improvement in blood glucose control and disease management in attendees following this program [24]. More specifically, a study in China has indicated the potential for directed educational programmes to result in improved knowledge of the impact of diabetes on bone health resulting in an increase in preventable behaviours in participants [17]. The internet and social media are often a means of information searching and learning for the public. Nevertheless, diabetes-related osteopathy is often not sufficiently emphasized in diabetes-focused public information websites, an important resource of information for individuals living with diabetes (Table 4). Interestingly, information about the link between diabetes and bone disease, as well as advice on how to improve bone health, is often easily accessible online at osteoporosis-related public websites, although these websites may not be often searched by individuals with diabetes, especially if they are not aware of diabetes-related osteopathy. Although it remains unclear whether nutrition and lifestyle interventions alone are sufficient to reduce fracture risk in a population with diabetes [5], it is a reasonable way to begin addressing this clinical concern.

To that end, the following is a list of proposed approaches that can be used to improve overall bone health in high-risk populations such as individuals living with diabetes:

- Educating and raising awareness among the public and patients about the risk of bone disease in high-risk individuals, including those living with diabetes.
- Training and educating healthcare professionals on best way of delivering bone health-related information and care, as they are often the primary source of diabetes-related information in people with diabetes (Table 4).
- Establishing systems to ensure that individuals with diabetes receive appropriate preventive, early-diagnostic and cost-effective treatment upon their level of risk.
- Promoting preventive lifestyle strategies including the development of health educational programs to address concerns and encourage a healthy lifestyle as well as intake of dietary calcium, vitamin D and weight-bearing exercise.
- Monitoring and evaluating bone health outcomes in a high-risk population through national screening and monitoring programs (e.g. bone mineral density assessment by dual x-ray absorptiometry).
- Improving co-disciplinary teamwork in delivering the best care possible (i.e., orthopaedics and endocrinologists).

It is acknowledged that this is not an easy task; that only multi-sectoral and coordinated responses with public policies and collaboration with the healthcare sector can fully address this matter.

### 4.2. Limitations of the Study

This study surveyed participants from a single diabetes centre in Galway, which may result in limited generalisability to domestic or international audiences. The care of individuals living with diabetes is standardized across Ireland, indicating generalisability across the country. Further, similar studies conducted in China [17] and Palestine [16] indicate the findings presented here remain valid internationally.

The study participants’ medical history (e.g., HbA_1C_ levels, specific medications) were not accessible due to ethical restrictions. The authors therefore could not validate a patient’s diabetic status, duration of diabetes, etc. However, the study participants were recruited within a diabetes clinic and would have received a clinical diagnosis of diabetes by a healthcare professional before enrolling in this clinical programme or participating in this study. The authors believe that these biochemical details were not critical to achieve the aim of this study, which is to investigate patients’ understanding and level of awareness of diabetes-related health complications such as diabetes-induced osteopathy. 

Finally, participants received an information leaflet prior to consent explaining the aim of this project. Within this leaflet the link of diabetes and bone was briefly explained. This leaflet by nature may have informed their responses, and in this case, the results from this study would represent an overestimation of the true awareness of diabetes-induced osteopathy. 

### 4.3. Study Conclusions

The aim of this study was to understand the current knowledge, perceptions, and concerns of individuals living with diabetes, specifically focusing on diabetes, its long-term health complications and association with osteopathy. Here, it was identified that the two cohorts of participants (those living with type 1 diabetes or type 2 diabetes) are distinct from one another regarding their concerns and knowledge of diabetes mellitus and its associated complications. These nuanced differences need to be considered when developing research or outreach programmes. Both cohorts identified nephropathy, amputations, and blindness as their greatest concerns, informing research programmes adhering to PPI principles. Concurrently, underappreciated diabetes-associated complications such as osteopathy should not be dismissed as these comorbidities are also underrepresented in educational material and potentially underrepresented when developing healthcare programmes when early intervention could have a significant positive impact on a patient’s long term quality of life.

## Figures and Tables

**Table 1:** Socio-demographic and clinical data of participants.

Participants’ Characteristics	Type 1 (*N* = 110)	Type 2 (*N* = 100)	*p*-Value
	*n* (%)	*n* (%)	
**Age group**			
19–29 years.	18 (16.6)	0 (0.0)	
30–39 years.	29 (26.8)	1 (1.0)	
40–49 years.	21 (19.4)	8 (8.0)	
50–59 years.	27 (25.0)	22 (22.0)	
60–69 years.	11 (10.2)	35 (35.0)	
70–79 years.	2 (1.85)	26 (26.0)	
80–89 years.	0 (0.0)	8 (8.0)	
*n* *	2	0	
Mean years. (SD)	43 (13.8)	64 (10.9)	<0.001
Range years.	[19–76]	[39–88]	
**Gender**			
Men	62 (56.4)	62 (62)	NS
Women	48 (43.6)	38 (38)	
**Irish county**			
Galway	83 (79.0)	76 (82.6)	
Mayo	12 (11.4)	10 (10.8)	
Other	10 (9.5)	6 (6.5)	
n*	5	8	
**Level of education**			
National School	3 (2.7)	25 (25.5)	<0.001
Irish Junior Cert	8 (7.3)	7 (7.1)	NS
Trade Apprenticeship	9 (8.2)	7 (7.1)	NS
Irish Leaving Cert	28 (25.5)	29 (29.6)	NS
Undergrad. Degree	33 (30.0)	13 (13.3)	0.002
Post-grad. Degree	21 (19.1)	9 (9.2)	0.037
Other	8 (7.3)	8 (8.2)	NS
n *	0	2	
**Years following diagnosis**	21 (13.3)	11 (6.0)	
mean (SD) and [range]	[<1–59]	[1–29]	<0.001

*N* = sample size; *n* = frequency, *n* * = missing, NS = not significant, SD = standard deviation. Statistics: 2-sample T-Test for ‘age’ and ‘years following diabetes diagnosis’ 2-Proportion Test for categorical data.

**Table 2:** Participants’ level of concern regarding developing potential diabetes-related health complications.

Participants’ Concerns	Type 1 *N* = 110	Type 2 *N* = 100	*p*-Value
	*n* (%)	*n* (%)	
**Level of concern:** median (IQR)	6.5 (4–8)	5.5 (5–7)	NS
**Analogue Scale:**			
[0–2] **No or little concerned**	7 (6.4)	15 (15.3)	0.038
[3–4] Somewhat concerned	22 (20)	8 (8.2)	0.012
[5] Rather concerned	15 (13.6)	26 (26.5)	0.020
[6–8] Moderately concerned	49 (44.5)	37 (37.7)	NS
[9–10] Extremely concerned	17 (15.4)	12 (12.2)	NS
n *	0	2	
**What concerned the participants the most?**			
Blindness	62 (68.8)	35 (43.2)	<0.001
Feet/amputation/circulation	37 (41.1)	40 (49.4)	NS
Kidney disease	24 (26.6)	8 (9.9)	0.003
Heart disease	15 (16.6)	8 (9.9)	NS
Future general health	10 (11.1)	12 (14.8)	NS
Nerve damage	6 (6.6)	4 (4.9)	+
Stroke/brain damage	9 (10.0)	4 (4.9)	+
Hypos/death/short-life	5 (5.5)	1 (1.2)	+
Pregnancy difficulties	3 (3.3)	0 (0.0)	+
Bone/osteoporosis	3 (3.3)	2 (2.5)	+
Infections	0 (0.0)	3 (3.7)	+
No concerns	4 (4.4)	11 (13.6)	0.037^+^
*n* *	20	19	

*N* = sample size; *n* = frequency, *n* * = missing, NS = not significant, IQR = interquartile range. Statistics: Mann Whitney 2 -sample T-Test for ‘level of concern’ (median). 2-Proportion Test for categorical data. (+) The normal approximation may be inaccurate for small samples.

**Table 3:** Respondents’ identification of potential diabetes-related health complications.

Patients’ disease knowledge	Type 1 (*N* = 110)	Type 2 (*N* = 100)	*p*-Value
	*n* (%)	*n* (%)	
**Total # of diabetes complications identified**	9 (6–11)	6 (4–9)	0.001
median (IQR)			
**Identification frequency**			
Retinopathy	99 (91.6)	82 (82.8)	NS
Nephropathy (dialysis and/or kidney damage)	90 (83.3)	62 (62.6)	0.001
Amputations and/or foot ulcers	87 (80.5)	69 (69.7)	NS
Heart disease	78 (72.2)	61 (61.6)	NS
Neuropathy	77 (71.3)	53 (53.5)	0.007
Stroke	71 (65.7)	57 (57.6)	NS
Increased infections	69 (63.8)	32 (32.3)	< 0.001
Liver failure	44 (40.7)	36 (36.4)	NS
Bone fractures	40 (37.0)	23 (23.2)	0.028
Pregnancy difficulties	31 (28.8)	7 (7.1)	< 0.001
Skin rashes	34 (31.5)	21 (21.2)	NS
Digestion difficulties	27 (25.0)	10 (10.1)	0.004
n *	2	1	

*N* = sample size, *n* = frequency, *n* * = missing, NS = not significant, IQR = interquartile range. Statistics: Mann Whitney 2-sample T-Test for ‘total complications identified’ (median). 2-Proportion Test for categorical data.

**Table 4:** Participants’ principal source of diabetes-related information.

Source of Information	Type 1 *N* = 110	Type 2 *N* = 100	*p*-Value
	*n* (%)	*n* (%)	
**Diabetes specialist**	90 (87.4)	56 (60.2)	<0.0001
**Internet/TV media**	37 (35.9)	23 (24.7)	NS
**Family doctor**	20 (19.4)	57 (61.3)	<0.0001
**Ophthalmologist**	26 (25.2)	24 (25.8)	NS
**Books/magazines**	17 (16.5)	21 (22.6)	NS
**Podiatrist**	11 (10.7)	16 (17.2)	NS
**Diabetes support groups**	9 (8.7)	9 (9.6)	NS
**Diabetes/community nurse**	8 (7.7)	6 (6.4)	NS
**Family/friends**	0 (0.0)	4 (4.3)	+
**Dietician**	2 (1.9)	0 (0.0)	+
*n* *	7	7	

*N* = sample size; *n* = frequency, *n* * = missing, NS = not significant. Statistics: 2-Proportions Test for categorical data. (+) the normal approximation may be inaccurate for small samples.

## Supplementary Materials

The following are available online at https://www.mdpi.com/2227-9032/8/1/25/s1, Supplementary S1: Level of education with participant’s age; Supplementary S2: Participants’ perceptions and understanding of diabetes; Supplementary S3: Evaluating participants’ level of concern with age and years following diagnosis with diabetes; Supplementary S4: Participants’ understanding of diabetic osteopathy.
