# Supplementary material for: Knowledge, Perceptions and Concerns of Diabetes-Associated Complications among Individuals Living with Type 1 and Type 2 Diabetes Mellitus
Source: Healthcare (Basel). 2020 Jan 30;8(1):25. doi: 10.3390/healthcare8010025 (PMC7151172; doi:10.3390/healthcare8010025)
Supplement: Supplementary file 1 [file healthcare-08-00025-s001.pdf]

healthcare

MDPI

Article

# Knowledge, Perceptions, and Concerns of Diabetes Mellitus and Its Associated Complications among Individuals Living with Type 1 and Type 2 Diabetes Mellitus

**Supplementary S1: Level of education with participant's age**

The participants' level of education was further subdivided by age (range of 10 years' intervals from 19 to 89 years). Data is presented as frequency (n). A significant percentage of individuals with type 2 diabetes (25.5%) had exited school with a primary level of education compared to 2% in the type 1 diabetes group (from which 24/25, and 2/2, were ≥ 60 years old, respectively). In contrast, more individuals with type 1 diabetes had pursued further education, including 30% pursuing an undergraduate degree (from which 30/33 were ≤ 60 yrs.) and 19% a post-graduate degree (from which 19/20 were ≤ 60 yrs.) compared to 13% (from which 7/13 were ≤ 60 yrs.) and 9% (from which 3/9 were ≤ 60 yrs.) in the type 2 diabetes group, respectively. Thus, the education level differences observed are likely due to the difference in age among both groups.

| **Type 1 Diabetes** | **Age Range (years)** | | | | | | | |
|---|---|---|---|---|---|---|---|---|
| **Level of education** | **19-29** | **30-39** | **40-49** | **50-59** | **60-69** | **70-79** | **80-89** | TOTAL |
| National school | 0 | 0 | 0 | 0 | 1 | 1 | 0 | 2 |
| Irish junior certificate | 0 | 0 | 2 | 4 | 2 | 0 | 0 | 8 |
| Trade apprenticeship | 0 | 4 | 0 | 2 | 3 | 0 | 0 | 9 |
| Irish leaving certificate | 7 | 7 | 6 | 6 | 1 | 1 | 0 | 28 |
| Undergraduate | 6 | 10 | 5 | 9 | 3 | 0 | 0 | 33 |
| Post-graduate | 3 | 6 | 6 | 4 | 1 | 0 | 0 | 20 |
| Other | 2 | 2 | 2 | 2 | 0 | 0 | 0 | 8 |
| TOTAL | 18 | 29 | 21 | 27 | 11 | 2 | 0 | 108 |

| **Type 2 Diabetes** | **Age Range (years)** | | | | | | | |
|---|---|---|---|---|---|---|---|---|
| **Level of education** | **19-29** | **30-39** | **40-49** | **50-59** | **60-69** | **70-79** | **80-89** | TOTAL |
| National school | 0 | 0 | 0 | 1 | 7 | 11 | 6 | 25 |
| Irish junior certificate | 0 | 0 | 1 | 3 | 2 | 1 | 0 | 7 |
| Trade apprenticeship | 0 | 0 | 0 | 2 | 4 | 1 | 0 | 7 |
| Irish leaving certificate | 0 | 0 | 2 | 7 | 13 | 6 | 1 | 29 |
| Undergraduate | 0 | 1 | 2 | 4 | 4 | 1 | 1 | 13 |
| Post-graduate | 0 | 0 | 1 | 2 | 2 | 4 | 0 | 9 |
| Other | 0 | 0 | 1 | 3 | 3 | 1 | 0 | 8 |
| TOTAL | 0 | 1 | 7 | 22 | 35 | 25 | 8 | 98 |

**Supplementary S2: Participants' perceptions and understanding of diabetes.**

Participants were asked to define and/or give phrases associated with diabetes in an open-ended question. Following is a selection of example responses for each category.

**Responses from individuals with type 1 diabetes**

- Definition of diabetes included words such as 'pancreas' and 'insulin' (74.5%): 'When the pancreas stops producing the hormone insulin' (#13, man, 20 yrs.). 'Deficiency at the pancreas

to produce insulin to enable energy release' (#39, woman, 35 yrs.). 'Diabetes is a consequence of your body not being able to produce insulin; as a result, insulin has to be given via injections' (#70, man, 40 yrs.).

- Definition of diabetes included words such as 'blood sugar', 'sugar control', 'glucose' (38.2%): 'Body's inability to regulate blood glucose resulting in hyperglycaemia or hypoglycaemia' (#43, man, 36 yrs.). 'Pancreas not producing insulin that is needed to enable sugar get into the muscles as energy' (#16, man, 50 yrs.). 'Low and high blood sugars' (#19, man, 31 yrs.)
- Definition of diabetes included words such as 'disease', 'condition', 'chronic disease', 'illness', 'disorder' (24.5%): 'Diabetes is a condition where a person's body stops producing insulin and needs to take insulin injections to substitute for this' (#56, man, 50 yrs.). 'Diabetes is a chronic, life-long, life-altering, life-threatening illness' (#4, woman, 55 yrs.).
- Definition of diabetes included technical words such as 'beta cells', 'Islets of Langerhans', 'autoimmune', 'immune reaction', 'immune attack' (11%): 'A T-cell autoimmune condition which inappropriately attacks beta cells of the pancreas' (#47, man, 30 yrs.). 'An autoimmune disease where the immune system attacks the pancreas (Islets of Langerhans) and no insulin is produced […]' (#66, woman, 54 yrs.). 'Type 1 diabetes is where the immune system attacks betta cells of the pancreas and results in the pancreas being unable to produce insulin. Insulin injections are essential for life' (#111, woman, 55 yrs.).
- Diabetes is defined as a challenge and/or it is associated with negative thoughts (9%): 'A pain in my…!' (#8, man, 44 yrs.). 'A disease that forces you to take control of your life and living' (#15, man, 45 yrs.). 'Annoying, hard-work, injections are a struggle' (#79, woman, 41 yrs.). 'Lifelong, frustrating' (#72, woman, 40 yrs.).
- Diabetes definition included both type 1 and type 2 diabetes (3.6%): 'In type 1 diabetes the pancreas no longer produces insulin; in type 2 diabetes the body cannot utilize insulin effectively' (#80, man, 44 yrs.). 'An inability to use insulin (type 2 diabetes), or an inability to make insulin (type 1 diabetes)' (#144, woman, 27 yrs.).
- Definition of diabetes included health complications of diabetes (1.8%), however no specific complications were named: 'Type 1 diabetes is a lack of insulin production resulting in need for injections to maintain sugar balance and a cause of further complications and health issues' (#14, man, 44 yrs.). 'If you don't look after your diabetes you will get complications' (#73, man, 43 yrs.).
- Diabetes is defined as having no disadvantages and/or concerns (0%). No responses of this type were found.

**Responses from individuals with type 2 diabetes**

- Definition of diabetes included words such as 'pancreas' and 'insulin' (19%): 'A inability to produce insulin in the pancreas' (#82, man, 62 yrs.). 'The inability to produce sufficient insulin to regulate blood sugars in the body' (#8, man, 43 yrs.). 'Diabetes is when you don't produce enough insulin' (#24, woman, 58 yrs.).
- Definition of diabetes included words such as 'blood sugar', 'sugar control', 'glucose' (39%): 'Where your body can't handle sugars and you need medication to help regulate your sugar levels' (#67, man, 48 yrs.). 'High blood sugars' (#21, woman, 39 yrs.), 'A problem your body has with glucose' (#78, man, 73 yrs.).
- Definition of diabetes included words such as 'disease', 'condition', 'chronic disease', 'disorder', 'illness' (4%): 'A chronic disease connected with the levels of sugar in blood' (#12, man, 71 yrs.). 'It is a condition when your pancreas fails to produce enough insulin to process sugars and carbohydrates in your blood resulting in increased blood sugar levels' (#72, woman, 50 yrs.).
- Definition of diabetes included technical words such as 'beta cells', 'Islets of Langerhans', 'autoimmune', 'immune reaction', 'immune attack' (2%): 'Not enough production of insulin by beta cells' (#14, man, 59 yrs.). 'Diabetes is that the pancreas – Islets of Langerhans – is not able to produce enough insulin which is needed' (#58, woman, 60 yrs.).

- Diabetes is defined as a challenge and/or it is associated with negative thoughts (17%): 'It's a pain, I can't eat nice things' (#40, woman, 71 yrs.). 'I'm tired, with no energy' (#25, man, 61 yrs.). 'Having to prick my finger 3 times a day and watching secret sugars in food' (#59, woman, 67 yrs.). 'I feel dizzy, I can't sleep, it's not easy' (#83, woman, 65 yrs.). 'Painful' (#89, man, 50 yrs.). 'It's terrible, not feeling well' (#99, woman, 56 yrs.). 'I can't get stabilized, one time I went hypoglycaemic, everything went black, it was scary' (#9, man, 74 yrs.).
- Diabetes definition included both type 1 and type 2 diabetes (0%).
- Definition of diabetes include health complications of diabetes such as damage of 'kidney', 'eyes', 'foot ulcers', 'amputations' and 'limbs' (7%): 'High blood sugar levels, frequent urination, thirst, complications from diabetes such as kidney, eye damage and foot ulcers' (#45, man, 55 yrs.). 'It is a disease to do with sugar levels and blood, and it affects feet, eyes, heart and other organs as well' (#97, man, 76 yrs.). '[…] it affects blood circulation and causes loss of limbs and eyesight' (#16, man, 67 yrs.).
- Diabetes is defined as having no disadvantages and/or concerns (11%): 'I have a normal life, I'm not too concern about it' (#38, man, 66 yrs.). 'I don't find any disadvantage, I have a healthy diet' (#84, man, 75yrs.). 'It's quite alright, I'm not too concern, I have it under control' (#31, woman, 85 yrs.).

**Supplementary S3: Multivariate linear regression models.**

**Multivariate linear regression analysis of participant's level of concern with age, years since diagnosis, gender and level of education.**

| | **Type 1 diabetes** | | **Type 2 diabetes** | |
|---|---|---|---|---|
| | **$\beta$ (95% CI)** | ***P*-value** | **$\beta$ (95% CI)** | ***P*-value** |
| **Age** | -0.035 (-0.078, 0.008) | 0.113 | -0.045 (-0.102, 0.013) | 0.126 |
| **Years since diagnosis** | 0.031 (-0.009, 0.070) | 0.126 | 0.125 (0.035, 0.215) | 0.007 |
| **Gender:** | | | | |
| **Male** | -0.876 (-1.851, 0.100) | 0.078 | 0.151 (-1.037, 1.338) | 0.801 |
| **Level of education:** | | | | |
| **National school** | -3.53 (-7.23, 0.17) | 0.061 | 0.56 (-1.83, 2.96) | 0.641 |
| **Trade apprenticeship** | 0.24 (-2.05, 2.52) | 0.839 | -0.06 (-2.86, 2.74) | 0.967 |
| **Irish leaving cert** | 0.097 (-1.84, 2.04) | 0.921 | 0.87 (-1.35, 3.09) | 0.438 |
| **Undergraduate** | 0.276 (-1.63, 2.18) | 0.775 | 0.33 (-2.10, 2.76) | 0.787 |
| **Post-graduate** | -0.65 (-2.71, 1.40) | 0.529 | 0.20 (-2.48, 2.89) | 0.880 |
| **Other** | -0.38 (-2.77, 2.00) | 0.750 | 1.34 (-1.43, 4.11) | 0.338 |

*B* = regression coefficient; 95%CI = 95% confidence interval.

**Multivariate linear regression analysis of level of disease knowledge (i.e. total number of complications of diabetes identified by participants) with age, years since diagnosis, gender and level of education.**

| | Type 1 diabetes | | Type 2 diabetes | |
|---|---|---|---|---|
| | *β* (95% CI) | *P*-value | *β* (95% CI) | *P*-value |
| **Age** | 0.038 (-0,034, 0.109) | 0.297 | -0.064 (-0.137, 0.009) | 0.085 |
| **Years since diagnosis** | -0.022 (-0.087, 0.044) | 0.513 | 0.065 (-0.0500, 0.1801) | 0.264 |
| **Gender:** | | | | |
| **Male** | -1,210 (-2.831, 0.410) | 0.141 | 1.050 (-0.442, 2.543) | 0.165 |
| **Level of education:** | | | | |
| **National school** | -6.12 (-12.26, 0.03) | 0.051 | -0.60 (-3.63, 2.44) | 0.696 |
| **Trade apprenticeship** | 0.52 (-3.28, 4.32) | 0.786 | -1.55 (-5.12, 2.02) | 0.390 |
| **Irish living cert** | -0.65 (-3.87, 2.57) | 0.690 | 1.36 (-1.46, 4.18) | 0.339 |
| **Undergraduate** | 1.09 (-2.08, 4.26) | 0.498 | 1.87 (-1.24, 4.97) | 0.235 |
| **Post-graduate** | 1.18 (-2.23, 4.60) | 0.493 | 0.93 (-2.49, 4.35) | 0.589 |
| **Other** | 1.13 (-2.83, 5.09) | 0.573 | 1.4 (-2.13, 4.93) | 0.431 |

*β* = regression coefficient; 95%CI = 95% confidence interval.

**Supplementary S4: Participants' understanding about diabetes-related osteopathy**

Participants were then asked to describe in an open-ended question how they thought diabetes could impact their bone health. Following is a selection of example responses for each category.

**Responses from individuals with type 1 diabetes:**

From the 110 respondents living with type 1 diabetes, a total of 19 (17.3%) participants left this question unanswered. Interestingly, a significantly higher percentage of participants with type 1 diabetes (34.5%) were unsure or unaware of the link between diabetes and bone health: *'To be honest, I was not aware until today that diabetes can have an impact on bone health'* (#36, man, 38 yrs.). *'I have never heard anything about bone health, so I have not kept any account of this condition'* (#98, man, 52 yrs.). *'I never knew it [diabetes] could affect my bone health but now that I have no thyroid, I need to research what to do'* (#59, woman, 52 yrs.).

Many participants gave well-structured and scientific answers to how their bone health could be affected by the onset of diabetes. Participants' answers were grouped into 5 main categories. When participants mentioned two different mechanisms of diabetes affecting bone health in the same answer, the counts were included in the different categories independently (4.5% overlapping responses).

- <u>Increased healing times and/or decreased bone strength</u> (27.3%): 'Diabetics have a lower healing power, so a fracture may take longer to heal' (#65, woman, 47 yrs.). 'From what I have read, diabetics commonly have weaker bones and are prone to bone fractures, which would surely result in a lengthy healing time' (#21, man, 25 yrs.).
- <u>Weakened immune system and higher risk of infections</u> (8.2%): 'Diabetes is linked to having a weakened immune system, which may result in inability and/or increased duration required to heal bone fractures' (#43, man, 36 yrs.). 'Your immune system might be weakened preventing your ability to heal well, or it may give rise to further infections around the fracture. Also, if you have excess weight, pressure on fracture might increase healing period' (#74, woman, 32 yrs.).
- <u>Poor circulation</u> (4.5%): 'Complications of diabetes can cause poor circulation, therefore sending less blood to tissue around a wound or a fracture can cause healing to become slower than normal and possible result also in higher infection risk' (#51, man, 38 yrs.). 'Maybe the blood circulation around the bones is too high in sugars leading to unhealthy bone' (#31, woman, 44 yrs.).
- <u>Bone cells</u> (1.8%): 'I think any complications could be with the bone cells' (#67, woman, 51 yrs.). 'Hormonal dysregulation may affect osteoblast/osteoclast integrity' (#47, man, 30 yrs.).
- <u>Vitamin D</u> (4.5%): 'Not having enough vitamin D or calcium to protect your bones' (#78, man, 58 yrs.). 'Diabetes can inhibit absorption of vitamin D, which is needed for bone health and bone healing. Diabetes can slow healing of all injuries due to sugar fluctuations' (#111, woman, 55

yrs.). 'I was not aware of the link between bone health per se and diabetes, however, I am aware of vitamin D deficiencies in diabetes is more pronounced, and the role of vitamin D in regulating the immune responses' (#112, woman, 44 yrs.).

**Responses from individuals with type 2 diabetes:**

From the 100 respondents living with type 2 diabetes, a total of 21 (21%) participants did not respond to this question. Forty participants (40%) openly responded that they were unaware and/or had never heard before about diabetes potentially impacting their bone health. '*I didn't know about the link between diabetes and bone health*' (#9, man, 74 yrs.). '*I was not aware of this as a potential problem*' (#82, man, 62 yrs.). *'I was not aware of issues in bone and that it [diabetes] could lead into fractures from falls'* (#56, man 54 yrs.). '*I was not aware that it [diabetes] could affect my bone health*' (#73, woman, 48 yrs.). '*I was not aware, and I have osteopenia*' (#40, woman, yrs.).

The majority of participants that responded to this question made accurate hypotheses about how diabetes could impact their bone health. However, the variety of proposed hypotheses/themes in the type 2 diabetes cohort was notably lower compared to the type 1 diabetes cohort. Responses from the type 2 diabetes cohort were grouped in the three main categories:

- Increased healing times and/or decreased bone strength (26%): 'Diabetes slow the healing of wounds; therefore, it may also take longer to heal a fracture' (#4, man, 60 yrs.). 'Diabetes makes bones easy to fracture, it may take longer [to heal] than most of other fractures' (#59, woman, 67 yrs.). 'Diabetes could result in thinning bones and affect bone fracture easily' (#58, woman, 60 yrs.). 'It might be possibly a result in the decrease of bone density' (#72, woman, 50 yrs.). 'Due to thinning of the bone and less healing power' (#74, woman, 71 yrs.).
- Weakened immune system and higher risk of infections (5%): 'There is a risk of serious infections due to the bone taking a longer time to heal, and at risk of gangrene and subsequent amputations' (#85, man, 60 yrs.). 'The immune system is reduced, it may take longer to heal a fracture, also less bone strength' (#38, man, 66 yrs.).
- Poor circulation (2%): 'Diabetes affects healing length, also due to circulation problems associated with diabetes' (#42, man, 61 yrs.).
- Bone cells (0%): No responses of this type were found.
- Vitamin D (0%): No responses of this type were found.

© 2020 by the authors. Submitted for possible open access publication under the terms and conditions of the Creative Commons Attribution (CC BY) license (http://creativecommons.org/licenses/by/4.0/).
